# The efficacy and ocular safety following aflibercept, conbercept, ranibizumab, bevacizumab, and laser for retinopathy of prematurity: a systematic review and meta-analysis

**DOI:** 10.1186/s13052-023-01543-3

**Published:** 2023-10-09

**Authors:** Jing Chen, Qingfei Hao, Jing Zhang, Yanna Du, Haoming Chen, Xiuyong Cheng

**Affiliations:** Department of Neonatology, The First Affiliated Hospital of Zhengzhou University, Zhengzhou, 450000 China

**Keywords:** Aflibercept, Bevacizumab, Conbercept, Laser, Retinopathy of prematurity, Ranibizumab

## Abstract

**Background:**

Retinopathy of prematurity (ROP) is typically treated with laser photocoagulation and/or intravitreal anti-vascular endothelial growth factor (anti-VEGF). To the best of our knowledge, most systematic reviews have focused on comparing anti-VEGF against laser treatment while comparisons between different anti-VEGF agents are lacking. Thus, we conducted this meta-analysis to compare the efficacy and safety of different anti-VEGF agents or laser after primary ROP therapy.

**Methods:**

We conducted a comprehensive search across multiple databases up to November 2022. We included studies that used anti-VEGF or laser for ROP with comparable cohorts.

**Results:**

Overall, 44 studies were included in this meta-analysis. When comparing anti-VGEF with laser, we found that the anti-VEGF group had a significantly higher retreatment rate (RR = 1.56, 95%CI = [1.06, 2.31], *p* = 0.03), a longer time from treatment to retreatment (WMD = 5.99 weeks, 95%CI = [4.03, 7.95], *p* < 0.001), a lower retinal detachment rate (RR = 0.55, 95%CI = [0.30, 0.91], *p* = 0.02), higher spherical equivalent (WMD = 1.69D, 95%CI = [0.61, 2.77], *p* = 0.002), lower myopia rate (RR = 0.69, 95%CI = [0.50, 0.97], *p* = 0.03) and lower anisometropia rate (RR = 0.44, 95%CI = [0.29, 0.67], *p* = 0.0001). In comparisons between ranibizumab and bevacizumab, the intravitreal ranibizumab (IVR) group was associated with higher recurrence rate (RR = 2.02, 95%CI = [1.49, 2.73], *p* < 0.0001), higher retreatment rate (RR = 1.70, 95%CI = [1.17, 2.47], *p* = 0.0006), and lower high myopia rate (RR = 0.31, 95%CI = [0.12, 0.77], *p* = 0.01). Similarly, when compared to aflibercept and conbercept, the IVR cohort also demonstrated higher recurrence and retreatment rates. While no significant differences were observed in any of the variables included in the statistical analysis in the comparison between bevacizumab and aflibercept.

**Conclusions:**

Anti-VEGF was associated with higher retreatment and lesser incidence of myopia as compared to laser. Laser therapy was linked to more complications like retinal detachment and myopia. Ranibizumab exhibited higher recurrence and retreatment rates compared to bevacizumab, aflibercept, and conbercept.

**Supplementary Information:**

The online version contains supplementary material available at 10.1186/s13052-023-01543-3.

## Introduction

Retinopathy of prematurity (ROP) is a disease of the retinal vascular which is mainly related to the immaturity of fetal organs and low body mass. The characteristic fundus change of ROP is the presence of pathological neovascularization with fibrosis [1]. Among children, ROP is the leading cause of preventable blindness. Blencowe et al. [2] found that 184,700 preterm infants developed varying degrees of ROP in 2010, of which approximately 20,000 had severe visual impairment or even blindness and 12,300 had mild to moderate visual impairment.

During the past few decades, laser photocoagulation has been a common treatment for ROP. However, it is controversial to use laser treatment due to its side effects, including visual myopia, field loss, and retinal damage [3]. Nowadays, anti-vascular endothelial growth factor (VEGF) is emerging as a promising therapeutic option in the management of ROP [4]. The RAINBOW study [5], the largest and most notable randomized clinical trial, reported that ranibizumab might be superior to laser therapy in the treatment of ROP, with fewer unfavourable ocular outcomes than laser therapy.

Despite clinical reports on laser and anti-VEGF treatment of ROP increasing, there remain conflicting results in the literature as to the efficacy and safety of the two kinds of treatment. And to the best of our knowledge, most systematic reviews have focused on comparing anti-VEGF against laser treatment while comparisons between different anti-VEGF agents are lacking. Hence, we conducted this meta-analysis to explore relative differences of different anti-VEGF agents or laser after primary ROP therapy. To date, this meta-analysis represents the most comprehensive attempt to summarize published findings about this topic in pursuit of guiding clinical decision-making.

## Materials and methods

This systematic review was carried out and reported using the protocols of the preferred reporting items for systematic reviews and meta-analyses [6]. The protocol was registered on the International Prospective Register of Systematic Reviews (PROSPERO CRD42022375107).

### Search Strategy

Using suitable predefined search terms, a search of the literature was carried out by two researchers in Web of Science, PubMed, EMBASE, ClinicalTrials, and the Cochrane Library from database inception to November, 2022.

### Selection criteria

#### Inclusion criteria

1. randomized controlled trials (RCTs) or comparative non-randomized studies.
2. studies reporting on premature infants with ROP.
3. studies comparing the anti-VEGF agents with laser or another anti-VEGF agent as monotherapy for ROP
4. articles presenting separate efficacy (like the regression rate, recurrence rate, retreatment rate etc.) and/or safety ophthalmologic data (including the retinal detachment, vitreous hemorrhage, cataract, and other adverse events).
5. Published in English

#### Exclusion criteria

1. The research types were non-comparative or inappropriate comparison studies, Meta-analysis, systematic or narrative reviews, case reports, conference abstract, erratums, replies, editorials, letter to editors, commentaries, notes, irrelevant topic, animal experimental studies, and non‐English literature.
2. patients with vitreoretinal conditions other than ROP.
3. articles that did not report relevant efficacy and/or safety data for both groups.
4. studies with repeated data.
5. studies reporting on less than 5 eyes per study group.
6. articles with a follow-up time of less than 6 months.
7. full-text articles were not available.

### Study selection and data extraction

According to the inclusion criteria, two investigators (Jing Chen and Qingfei Hao) independently screened the appropriate articles and extracted the data. We selected the articles after mutual agreement of both authors. The following baseline characteristics and study outcomes were collected for each included study: study author and year, country, study design, single center or multicenter, follow-up time, sample size, birth weight, gestational age, postmenstrual age at treatment, time between treatment and retreatment, intervention (including the intravitreal injection agent and dose), type of ROP, regression number, recurrence number, retreatment number, eye complication number (like retinal detachment, vitreous hemorrhage, endophthalmitis, cataract), spherical equivalent (SE), cylinder, emmetropia and other interested outcomes.

### Study quality assessment

A risk of bias assessment was conducted independently by two reviewers (Jing Zhang and Yanan Du) for each trial. For randomized controlled trials, the Cochrane risk of bias tool was used [7]. Depending on each domain (sequence generation, concealment of allocation, masking of participants and outcome assessors, incomplete outcome data, selective reporting of outcomes and other potential threats to validity), the bias risk was classified as low, high, or unclear. As for non-randomized comparative studies, we used the Newcastle–Ottawa Scale (NOS) [8]. Potential disagreements that may have arisen during the data extraction procedure and quality assessment were resolved by discussion and agreement with a third author.

### Data analysis

Data were combined and analyzed using RevMan version 5.3 software (Cochrane Collaboration). The heterogeneity was quantified using the I^2^ and the *P*-value of the Chi-square test, where I^2^ > 50% and* P* value < 0.1 indicated substantial heterogeneity. For this Meta-analysis, we used a random-effect model when there was heterogeneity between studies. Otherwise, a fixed-effected model was presented [9]. Continuous parameters were presented as means ± standard deviations, while categorical data were expressed as percentages. Dichotomous variables were analyzed using risk ratios (RR) and weighted mean differences (WMDs) were used for continuous endpoints. And 95% confidence interval (CI) was included for all outcomes. Meanwhile, forest plots were drawn to demonstrate variation and to explore heterogeneity.

## Results

### Study selection and characteristics of eligible studies

**Fig. 1:**
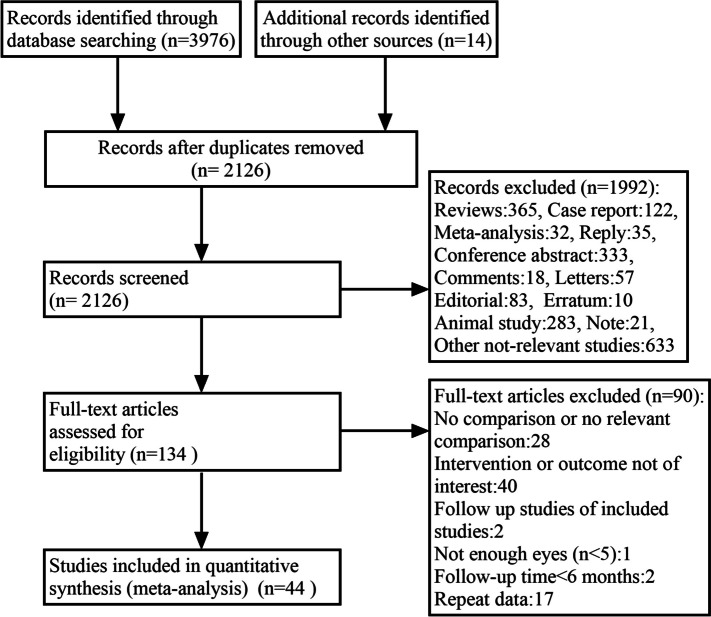
Flow diagram of literature search process

In Fig. 1, the literature screening process and results are displayed. In total, 3990 articles were retrieved through the literature search. We evaluated 2126 articles by reading titles and abstracts after excluding 1864 duplicate studies. Subsequently, 134 literatures were advanced to full-text screening, 90 of which were excluded. Finally, 44 articles were considered eligible for inclusion in the meta-analysis [3, 5, 10–51]. Each trial had a minimum follow-up period of six months. Among the 44 studies includeded, 8 were RCTs and 36 were nonrandomized studies. Of these, 30 studies compared anti-VEGF and laser therapy; 7 studies compared bevacizumab and ranibizumab; 1 study compared ranibizumab and aflibercept; 2 study compared bevacizumab and aflibercept; 3 study compared ranibizumab and conbercept and 1 study compared bevacizumab, ranibizumab, and aflibercept. Characteristics of included studies are shown in (Table S1, Additional file).

### Assessment of risk of bias and quality of evidence

A total of 8 studies were reviewed by the Cochrane risk of bias assessment for RCTs. Overall, most of included RCTs had a low risk of bias (Fig. S1, Additional file). In summary, there were 35 of 56 (62.5%) Cochrane domains showed low risk of bias, 7 (12.5%) unclear risk, and 14 (25%) high risk. The main concerns about bias were masking of participants and personnel and masking of outcome assessment.

Thirty-six non-randomized studies were assessed for risk of bias by the NOS (Table S2, Additional file). There was a low risk of bias in the majority of them, 237 of 288 (82.3%) NOS domains were linked to low risk domains and 51 (17.7%) had a high or unclear risk of bias. High risk of bias were mainly due to lack of demonstration that patients who had received any previous treatments before intravitreal injections were excluded.

### Outcomes

#### Anti-VEGF vs laser

##### Efficacy outcomes

**Fig. 2:**
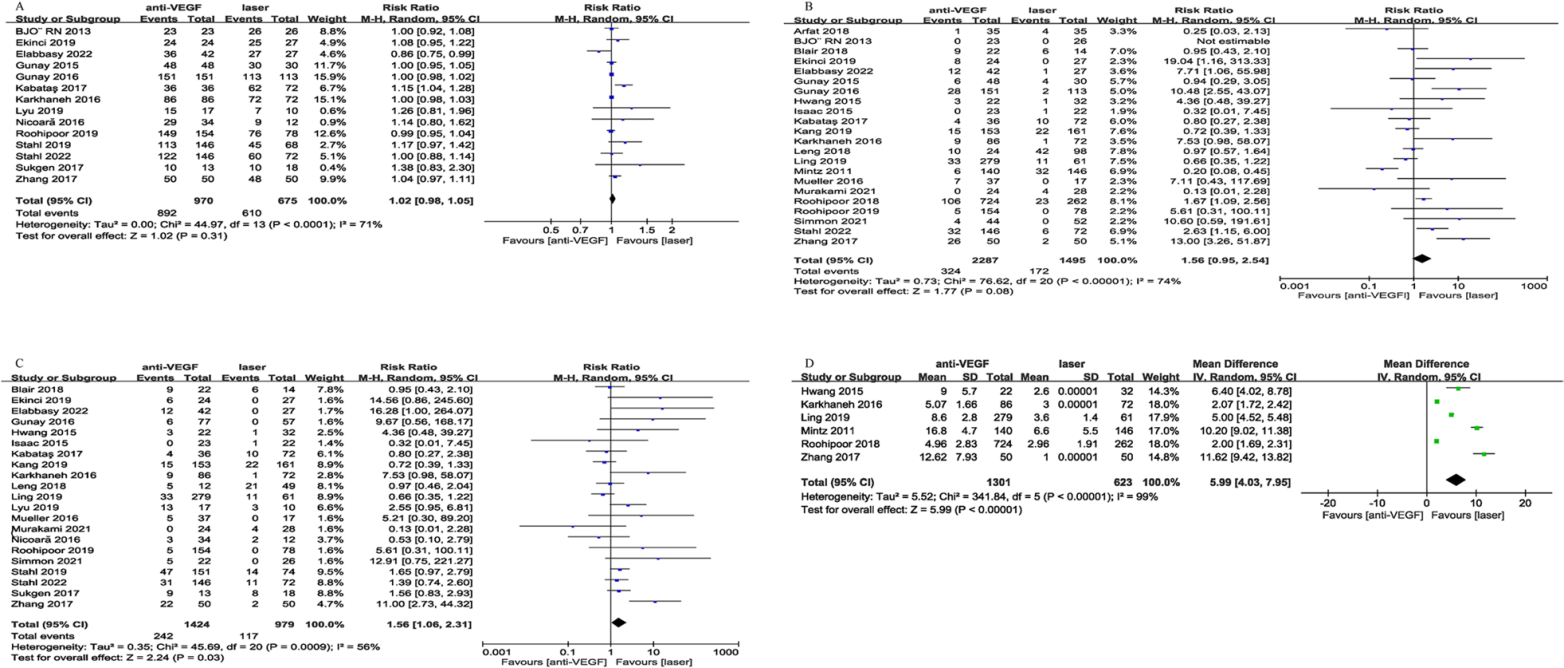
Forest plot of the efficacy outcomes between anti-VEGF and laser

In the main analysis, there was no significant difference in regression rate between anti-VEGF and laser (*p* = 0.31, Fig. 2A). Recurrence incidence also showed similar results (*p* = 0.08, Fig. 2B). Compared to laser, anti-VEGF group had a significantly higher retreatment rate (RR = 1.56, 95%CI = [1.06, 2.31], *p* = 0.03, Fig. 2C). In the anti-VEGF cohort, the time from treatment to retreatment was significantly longer than in the laser group (WMD = 5.99 weeks, 95%CI = [4.03, 7.95], *p* < 0.001, Fig. 2D).

##### Safety outcomes

**Fig. 3:**
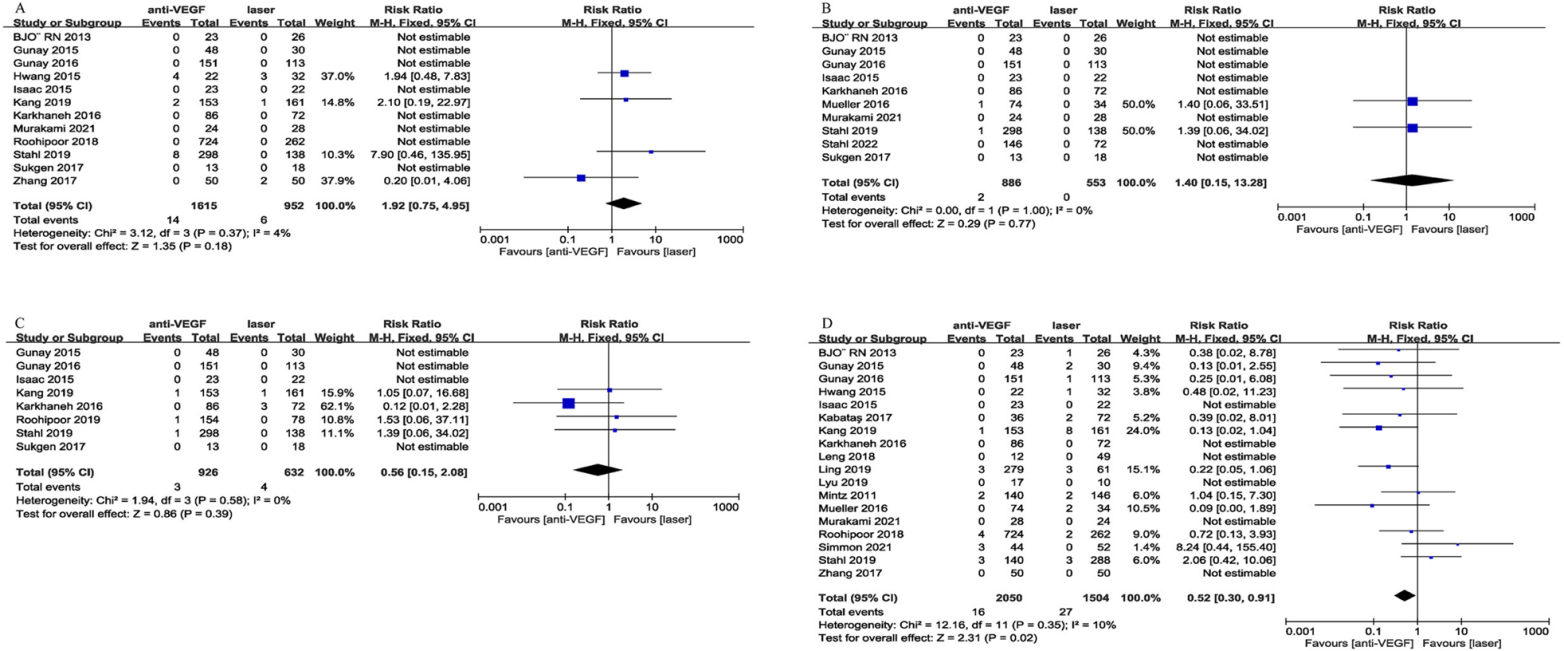
Forest plot of safety outcomes between anti-VEGF and laser

Except for retinal detachment, all analyzed complications did not show significant differences between the two groups, including vitreous hemorrhage (*p* = 0.18, Fig. 3A), endophthalmitis (*p* = 0.77, Fig. 3B), cataract (*p* = 0.39, Fig. 3C). However, the retinal detachment rate was significantly decreased in anti-VEGF relative to laser (RR = 0.55, 95%CI = [0.30, 0.91], *p* = 0.02, Fig. 3D).

##### Refractive outcomes

**Fig. 4:**
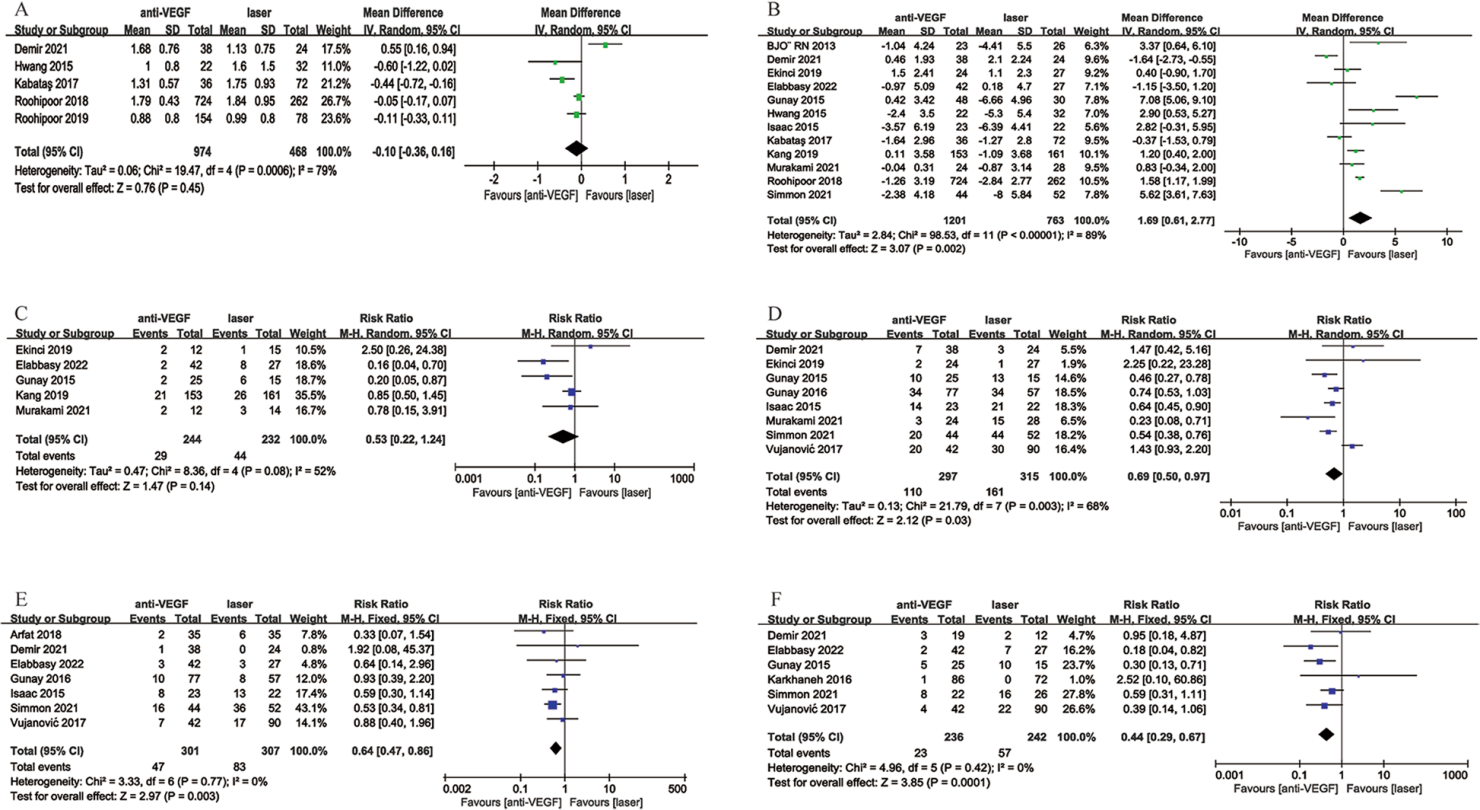
Forest plot of refractive outcomes between anti-VEGF and laser

Refractive outcomes were mixed as there was no significant difference for cylinder (*p* = 0.45, Fig. 4A); however, anti-VEGF cohort was associated with significantly higher spherical equivalent compare to laser (WMD = 1.69D, 95%CI = [0.61, 2.77], *p* = 0.002, Fig. 4B). There was no significant difference for strabismus (*p* = 0.14, Fig. 4C). There was a significantly lower proportion of myopia (RR = 0.69, 95%CI = [0.50, 0.97], *p* = 0.03, Fig. 4D) and high myopia (RR = 0.64, 95%CI = [0.47, 0.86], *p* = 0.003, Fig. 4E) in the anti-VEGF relative to laser. In addition, the rate of anisometropia was significantly lower in the anti-VEGF group compare to laser (RR = 0.44, 95%CI = [0.29, 0.67], *p* = 0.0001, Fig. 4F).

#### Comparisons between different anti-VEGF agents

##### Intravitreal Ranibizumab (IVR) VS Intravitreal Bevacizumab (IVB)

**Fig. 5:**
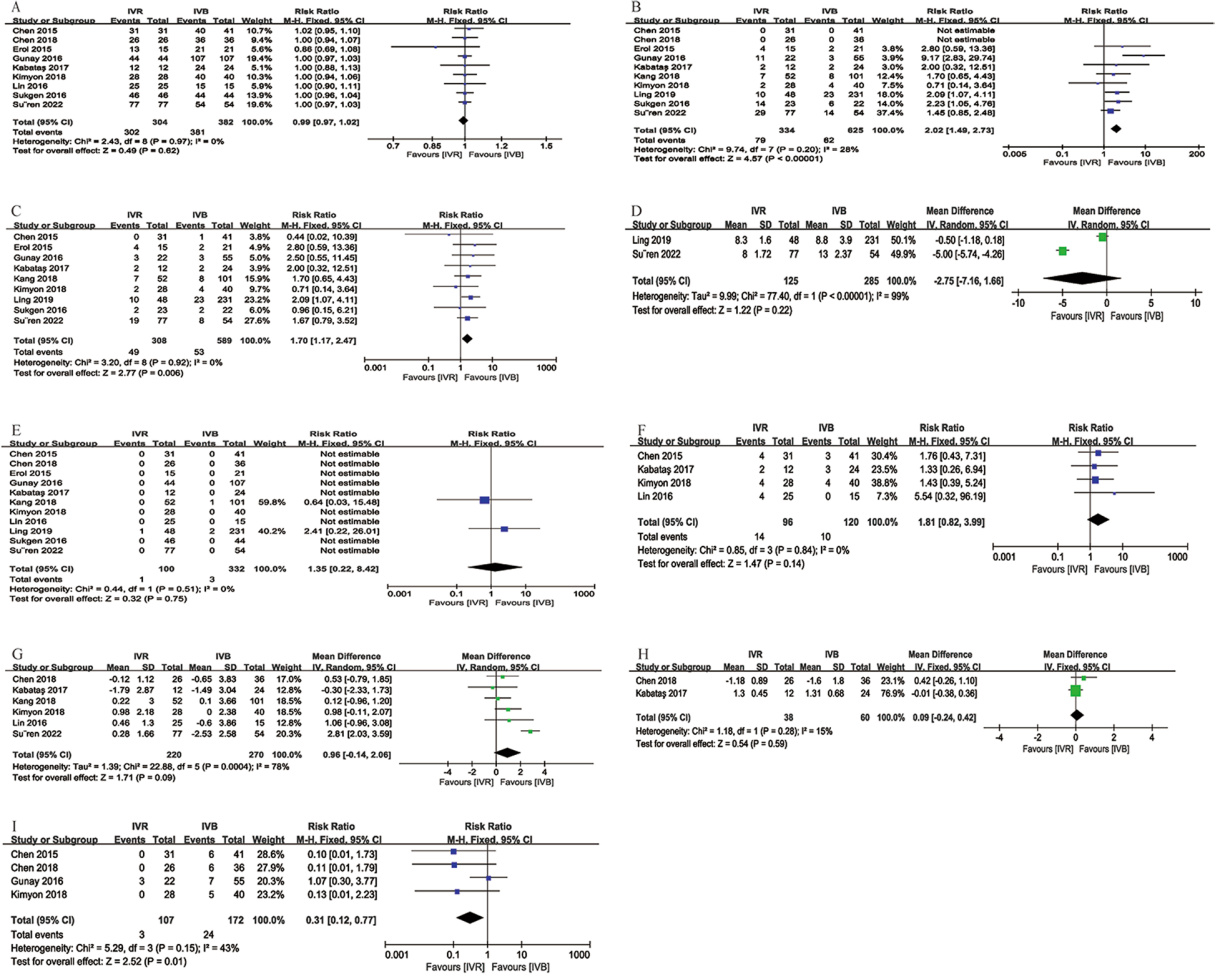
Comparison outcomes between IVR and IVB

There were 11 studies that compared IVR with IVB, the outcomes of the comparisons were as follows: regression rate (no significant difference, *p* = 0.62, Fig. 5A), recurrence rate (significantly lower in the IVB cohort, RR = 2.02, 95%CI = [1.49, 2.73], *p* < 0.0001, Fig. 5B), retreatment rate (significantly lower in the IVB group, RR = 1.70, 95%CI = [1.17, 2.47], *p* = 0.0006, Fig. 5C), time from treatment to retreatment (no significant difference, *p* = 0.22, Fig. 5D), retinal detachment rate (no significant difference, *p* = 0.75, Fig. 5E), preretinal hemorrhage (no significant difference, *p* = 0.14, Fig. 5F), spherical equivalent (no significant difference, WMD = 0.96D, 95%CI = [-0.14, 2.06], *p* = 0.09, Fig. 5G), cylinder (no significant difference, *p* = 0.59, Fig. 5H), high myopia (significantly lower proportion in the IVR group, RR = 0.31, 95%CI = [0.12, 0.77], *p* = 0.01, Fig. 5I).

##### IVR VS Intravitreal Conbercept (IVC)

When the comparisons were conducted between the IVR and IVC, the results were as follows: regression rate (no significant difference, *p* = 0.41, Fig. S2A, Additional file), recurrence rate (significantly lower in the IVC group, RR = 2.09, 95%CI = [1.70, 2.56], *p* < 0.0001, Fig. S2B, Additional file), retreatment rate (significantly lower in the IVC group, RR = 2.01, 95%CI = [1.53, 2.63], *p* < 0.0001, Fig. S2C, Additional file), time from treatment to retreatment (no significant difference, *p* = 0.13, Fig. S2D, Additional file).

##### IVB VS Intravitreal Aflibercept (IVA)

The outcomes of the comparisons between the IVB and IVA were as follows: regression rate (no significant difference, *p* = 0.62, Fig. S3A, Additional file), recurrence rate (no significant difference, *p* = 0.16, Fig. S3B, Additional file), retreatment rate (no significant difference, *p* = 0.35, Fig. S3C, Additional file), time from treatment to retreatment (no significant difference, *p* = 0.46, Fig. S3D, Additional file), spherical equivalent (no significant difference, *p* = 0.38, Fig. S3E, Additional file).

##### IVR VS IVA

Based on the comparisons between the IVR and IVA, the following results were obtained: regression rate (no significant difference, *p* = 1.0, Fig. S4A, Additional file), recurrence rate (significantly lower in the IVA cohort, RR = 2.33, 95%CI = [1.11, 4.91], *p* = 0.03, Fig. S4B, Additional file), retreatment rate (significantly lower in the IVA group, RR = 2.06, 95%CI = [1.16, 3.67], *p* = 0.01, Fig. S4C, Additional file), time from treatment to retreatment (significantly shorter in the IVR group, WMD = -5.03 weeks, 95%CI = [-6.99, -3.07], *p* < 0.0001, Fig. S4D, Additional file).

## Discussion

The present meta-analysis compared the efficacy and safety of intravitreal anti-VEGF to laser therapy and different anti-VEGF agents in the treatment of ROP. The pooled effect estimate showed laser treatment was associated with a significantly lower retreatment rate and shorter duration until retreatment. In terms of safety and refractive outcomes, anti-VEGF was associated with a lower retinal detachment rate, higher spherical equivalent, lower myopia rate, lower high myopia rate, and lower anisometropia rate. When compared IVR with IVB, IVR was associated with a higher recurrence rate and retreatment rate. Similarly, the recurrence rate and retreatment rate were significantly higher in the IVR cohort compared to IVC and IVA. Regarding several variables of efficacy, no significant difference was found between IVB and IVA.

### Anti-VEGF vs laser

When it came to some new therapies to treat diseases, the most critical considerations included not only efficacy but safety as well. In recent years, numerous studies have examined the efficacy and safety of anti-VEGF in comparison with laser treatment for ROP, but there is no consistency in the results. A recent multicenter RCT comparing aflibercept with laser photocoagulation demonstrated that treatment success rates of IVA and laser were 85.5% and 82.1% respectively, and rescue treatment was required in 4.8% of eyes in the IVA group vs 11.1% in the laser photocoagulation group [45]. Another study by Stahl et al. [5] concluded that ranibizumab was as effective and safe as laser therapy in treating ROP, and could potentially be superior. And a meta-analysis of 3701 eyes found that anti-VEGF was associated with a significantly higher retreatment rate and a longer time from treatment to retreatment, however, there was no significant difference in the regression rate between anti-VEGF and laser [52]. Our findings are exactly consistent with these in terms of efficacy, which were also in line with Zhang et al.’s study [51].

With respect to safety outcomes, some studies reported the potential risk of complications like retinal detachment, visual field loss, and cataracts after laser treatment [53, 54]. A meta-analysis by Taher et al. [55] suggested that anti-VEGF injections were associated with significantly fewer adverse events than laser in general. However, Popovic et al. [52] found there was no significant difference between anti-VEGF and laser for all analyzed complications, including vitreous hemorrhage, retinal detachment, and retinal hemorrhage. Recently, the effects of anti-VEGF on neurodevelopmental outcomes have also received increasing attention [56]. In the present study, we found no significant differences in all analyzed complications between the anti-VEGF and laser groups except for retinal detachment. The retinal detachment rate was significantly decreased in anti-VEGF relative to laser, which is in line with the study by Barry et al. [57]. The underlying mechanisms contributing to the increased likelihood of retinal detachment following laser treatment are not fully understood. This phenomenon may be related to the quicker action of anti-VEGF therapy or potential anterior segment damage caused by laser [57, 58].

As for the refractive outcomes, our results showed that the anti-VEGF cohort was associated with a significantly higher spherical equivalent, which is consistent with Kong et al. [59]. However, Popovic et al. and Simmons et al. both found no significant difference in SE [44, 52]. This discrepancy could be attributed to the small sample size and short follow-up duration. Additionally, the anti-VEGF group displayed a significantly lower myopia rate, high myopia rate, and anisometropia rate than the laser group. The studies of Tsiropoulos et al. and Tan et al. supported this conclusion [60, 61].

### Comparisons between different anti-VEGF agents

In the current treatment of ROP, ranibizumab, bevacizumab, aflibercept, and conbercept are the main drugs. However, systematic reviews focused on comparing different anti-VEGF agents are lacking. Only one meta-analysis by Chang et al. [62] compared the effectiveness of different anti-VEGF drugs, they found that in terms of retreatment rates, bevacizumab ranked first, followed by aflibercept second, and ranibizumab last. In the present study, we also found that IVR was associated with a higher recurrence rate and retreatment rate when IVB, IVA, and IVC. This may partially be influenced by differences in the intraocular half-lives of drugs [63]. Previous studies have hypothesized that IVR may be associated with a higher recurrence rate than IVB, IVA, and IVC due to its shorter half-life [10, 20, 27, 47]. And, there was no significant difference in complications and ocular refractive results between IVB and IVR.

This meta-analysis has certain limitations. First of all, a combination of nonrandomized cohort studies and RCTs was conducted, which could increase the risk of bias. Second, there was heterogeneity in some variables. This is an expected feature when nonrandomized trials were included, and we minimized this problem by using a random-effects model for this meta-analysis. Third, fewer studies were included when comparing differences between anti-VEGF agents, which may cause the statistical power to detect a difference to be limited.

## Conclusion

Overall, anti-VEGF was associated with higher retreatment and lesser incidence of myopia as compared to laser. Laser therapy was linked to more complications like retinal detachment and myopia. Ranibizumab exhibited higher recurrence and retreatment rates compared to bevacizumab, aflibercept, and conbercept. There is a need for more high-quality RCTs to be conducted before formal clinical recommendations can be made regarding the superiority of anti-VEGF agents or laser therapy in the clinical practice of treating ROP.

### Supplementary Information

**Additional file 1: Table S1.** Characteristics of the included studies. **Fig. S1.** Risk‐of‐bias summary of the included randomized controlled trial. **Table S2.** Newcastle-Ottawa Risk of Bias Assessment of Non-Randomized Studies. **Fig. S2.** Comparison outcomes between IVR and IVC. **Fig. S3.** Comparison outcomes between IVB and IVA. **Fig. S4.** Comparison outcomes between IVR and IVA.

## Data Availability

All data generated or analysed during this study are included in this published article and its supplementary information files.

## Abbreviations

ROP: Retinopathy of prematurity
VEGF: Vascular endothelial growth factor
RCT: Randomized controlled trial
IVR: Intravitreal ranibizumab
IVB: Intravitreal bevacizumab
IVA: Intravitreal aflibercept
IVC: Intravitreal conbercept
SE: Spherical equivalent
NOS: Newcastle–Ottawa Scale
RR: Risk ratios
WMD: Weighted mean difference
CI: Confidence interval
