# Supplementary material for: The efficacy and ocular safety following aflibercept, conbercept, ranibizumab, bevacizumab, and laser for retinopathy of prematurity: a systematic review and meta-analysis
Source: Ital J Pediatr. 2023 Oct 9;49:136. doi: 10.1186/s13052-023-01543-3 (PMC10561404; doi:10.1186/s13052-023-01543-3)
Supplement: Supplementary file 1 — Additional file 1: Table S1. Characteristics of the included studies. Fig. S1. Risk‐of‐bias summary of the included randomized controlled trial. Table S2. Newcastle-Ottawa Risk of Bias Assessment of Non-Randomized Studies. Fig. S2. Comparison outcomes between IVR and IVC. Fig. S3. Comparison outcomes between IVB and IVA. Fig. S4. Comparison outcomes between IVR and IVA. [file 13052_2023_1543_MOESM1_ESM.docx]

**Search Strategy:** We searched with the terms “Bevacizumab” OR “Mvasi” OR “Avastin” OR “Ranibizumab” OR “Lucentis” OR “Aflibercept” OR “Conbercept” OR “anti-VEGF” OR “Angiogenesis Inhibitors” OR “anti-vascular endothelial growth factor” AND “Retinopathy of Prematurity” OR “Prematurity Retinopathies” OR “Prematurity Retinopathy” OR “Retrolental Fibroplasia” OR “Fibroplasia, Retrolental” OR “Fibroplasias, Retrolental” OR “Retrolental Fibroplasias” OR “ROP”

Table S1 Characteristics of the included studies

| Study | Study design | Invention | Recurrence rate (%) | Retreatment rate (%) | Time from treatment to retreatment (weeks) | Other outcomes |
| --- | --- | --- | --- | --- | --- | --- |
| Arfat 2018 | NRS | IVB (0.625mg) vs laser | 2.9 vs 11.4 | NA | NA | Incidence of high myopia |
| Bjorn 2013 | NRS | IVB (0.375mg or 0.625mg) vs laser | 0 vs 0 | NA | NA | Incidence of regression, retinal detachment, vitreous hemorrhage, endophthalmitis, SE |
| Blair 2018 | NRS | IVB (0.5-0.625mg) vs laser | 40.9 vs 42.9 | 40.9 vs 42.9 | NA | NA |
| Chen 2015 | NRS | IVR (0.25mg) vs IVB (0.625mg) | 0 vs 0 | 0 vs 2.4 | NA | Incidence of regression, retinal detachment, preretinal hemorrhage, high myopia |
| Chen 2018 | NRS | IVR (0.25mg) vs IVB (0.625mg) | 0 vs 0 | NA | NA | Incidence of regression, retinal detachment, high myopia, SE |
| Cheng 2020 | NRS | IVR (0.25mg) vs IVC (0.25mg) | 30.6 vs 15.5 | 30.5 vs 15.5 | 8.1 vs 11.0 | Incidence of regression |
| Demir 2021 | NRS | IVB (0.625mg) vs laser | NA | NA | NA | Incidence of anisometropia, myopia, high myopia, SE, cylinder power |
| Ekinci 2019 | NRS | IVA (1mg) vs laser | 33.3 vs 0 | 25 vs 0 | NA | Incidence of regression, strabismus, myopia, SE |
| Elabbasy 2022 | NRS | IVR (0.25mg) vs laser | 28.6 vs 3.7 | 28.6 vs 0 | NA | Incidence of regression, strabismus, anisometropia, high myopia, SE |
| Erol 2015 | NRS | IVR (0.25mg) vs IVB (0.625mg) | 26.7 vs 9.5 | 26.7 vs 9.5 | NA | Incidence of regression, retinal detachment |
| Geloneck 2014 | RCT | IVB (0.625mg) vs laser | NA | NA | NA | Incidence of high myopia, SE |
| Gunay 2015 | NRS | IVB (0.625mg) vs laser | 12.5 vs 13.3 | NA | NA | Incidence of regression, retinal detachment, vitreous hemorrhage, endophthalmitis, cataract, strabismus, anisometropia, myopia, SE |
| Gunay 2016 | NRS | IVR (0.25mg) vs IVB (0.625mg) vs laser | 50 vs 5.5 vs 1.8 | 13.6 vs 5.5 vs 0 | NA | Incidence of regression, retinal detachment, vitreous hemorrhage, endophthalmitis, cataract, myopia, high myopia |
| Hwang 2015 | NRS | IVB (0.625mg) vs laser | 13.6 vs 3.1 | 13.6 vs 3.1 | 9.0 vs 2.6 | Incidence of retinal detachment, vitreous hemorrhage, SE, cylinder power |
| Isaac 2015 | NRS | IVB (0.625mg) vs laser | 0 vs 4.6 | 0 vs 4.6 | NA | Incidence of retinal detachment, vitreous hemorrhage, endophthalmitis, cataract, myopia, high myopia, SE |
| Jin 2017 | NRS | IVR (0.25mg) vs IVC (0.25mg) | 39.3 vs 0 | 39.3 vs 0 | NA | Incidence of regression |
| Kabatas 2017 | NRS | IVR (0.25mg) vs IVB (0.625mg) vs laser | 16.7 vs 8.3 vs 13.9 | 16.7 vs 8.3 vs 13.9 | NA | Incidence of regression, retinal detachment, preretinal hemorrhage, SE, cylinder power |
| Kang 2018 | NRS | IVR (0.2mg) vs IVB (0.625mg) | 13.5 vs 7.9 | 13.5 vs 7.9 | NA | Incidence of retinal detachment, SE |
| Kang 2019 | NRS | IVR (0.25mg) vs laser | 9.8 vs 13.7 | 9.8 vs 13.7 | NA | Incidence of retinal detachment, vitreous hemorrhage, cataract, strabismus, SE |
| Karkhaneh 2016 | RCT | IVB (0.625mg) vs laser | 10.5 vs 1.4 | 10.5 vs 1.4 | 5.07 vs 3.0 | Incidence of regression, retinal detachment, vitreous hemorrhage, endophthalmitis, cataract, anisometropia |
| Kimyon 2018 | NRS | IVR (0.25mg) vs IVB (0.625mg) | 7.1 vs 10.0 | 7.1 vs 10.0 | NA | Incidence of regression, retinal detachment, preretinal hemorrhage, high myopia, SE |
| Leng 2018 | NRS | IVR (0.25mg) vs laser | 41.7 vs 42.9 | 41.7 vs 42.9 | NA | Incidence of retinal detachment, |
| Lin 2016 | NRS | IVR (0.25mg) vs IVB (0.625mg) | NA | NA | NA | Incidence of regression, retinal detachment, preretinal hemorrhage, SE |
| Ling 2019 | NRS | IVR (0.25mg) vs IVB (0.625mg) vs laser | 20.8 vs 10.0 vs 18.0 | 20.8 vs 10.0 vs 18.0 | 8.3 vs 8.8 vs 3.6 | Incidence of retinal detachment, |
| Linghu 2022 | NRS | IVR (0.25mg) vs IVC (0.25mg) | 29.3 vs 13.4 | NA | NA | Incidence of regression |
| Lyu 2019 | NRS | IVR (0.25mg) vs laser | NA | 76.5 vs 30.0 | NA | Incidence of regression, retinal detachment, |
| Milan 2022 | NRS | IVB (0.625mg) vs IVA (1mg) | 1.9 vs 15.8 | 1.9 vs 15.8 | 9.1 vs 15.5 | Incidence of regression |
| Mintz 2011 | RCT | IVB (0.625mg) vs laser | 4.3 vs 21.9 | NA | 16.8 vs 6.6 | Incidence of retinal detachment, |
| Mueller 2016 | NRS | IVB (0.625mg) vs laser | 18.9 vs 0 | 13.5 vs 0 | NA | Incidence of retinal detachment, endophthalmitis |
| Murakami 2021 | NRS | IVB (0.625mg) vs laser | 0 vs 14.3 | 0 vs 14.3 | NA | Incidence of retinal detachment, vitreous hemorrhage, endophthalmitis, strabismus, myopia, SE |
| Nicoară 2016 | NRS | IVB (0.625mg) vs laser | NA | 8.8 vs 16.7 | NA | Incidence of regression |
| Riazi 2021 | NRS | IVB (0.625mg) vs IVA (1mg) | 3.9 vs 58.3 | NA | NA | Incidence of regression |
| Roohipoor 2018 | RCT | IVB (0.625mg) vs laser | 14.6 vs 8.8 | NA | 4.9 vs 2.9 | Incidence of retinal detachment, vitreous hemorrhage, SE, cylinder power |
| Roohipoor 2019 | NRS | IVB (0.625mg) vs laser | 3.25 vs 0 | 3.25 vs 0 | NA | Incidence of regression, cataract, cylinder power |
| Simmon 2021 | NRS | IVB (0.625mg) vs laser | 9.1 vs 0 | 22.73 vs 0 | NA | Incidence of retinal detachment, anisometropia, myopia, high myopia, SE |
| Stahl 2019 | RCT | IVR (0.2mg) vs laser | NA | 31.1 vs 18.9 | NA | Incidence of regression, retinal detachment, vitreous hemorrhage, endophthalmitis, cataract |
| Stahl 2022 | RCT | IVA (0.4mg) vs laser | 21.9 vs 8.3 | 21.2 vs 15.3 | NA | Incidence of regression, endophthalmitis |
| Sukgen 2016 | NRS | IVR (0.25mg) vs IVB (0.625mg) | 60.9 vs 27.3 | 8.7 vs 9.1 | NA | Incidence of regression, retinal detachment |
| Sukgen 2017 | NRS | IVR (0.25mg) vs laser | NA | 69.2 vs 44.4 | NA | Incidence of regression, vitreous hemorrhage, endophthalmitis, cataract |
| Sukgen 2018 | NRS | IVR (0.25mg) vs IVA (1mg) | 48.1 vs 13.9 | 22.2 vs 8.3 | 8.2 vs 14.2 | Incidence of regression |
| Su¨ren 2022 | NRS | IVR (0.25mg) vs IVB (0.625mg) vs IVA (1mg) | 37.7 vs 25.9 vs 23.2 | 24.7 vs 14.8 vs 14.3 | 8 vs 13 vs 12 | Incidence of regression, retinal detachment, SE |
| Vujanović 2017 | NRS | IVB (0.625mg) vs laser | NA | NA | NA | Incidence of anisometropia, myopia, high myopia |
| Wu 2021 | RCT | IVR (0.25mg) vs IVC (0.25mg) | 23.3 vs 16.7 | 23.3 vs 16.7 | 8.3 vs 8.7 | NA |
| Zhang 2017 | RCT | IVR (0.3mg) vs laser | 52.0 vs 4.0 | 44.0 vs 4.0 | 12.6 vs 1 | Incidence of regression, retinal detachment, vitreous hemorrhage |

RCT: randomized controlled trial; NRS: non-randomized controlled study; IVR: intravitreal ranibizumab; IVB: intravitreal bevacizumab; IVA: intravitreal aflibercept; IVC: intravitreal conbercept; SE: spherical equivalent


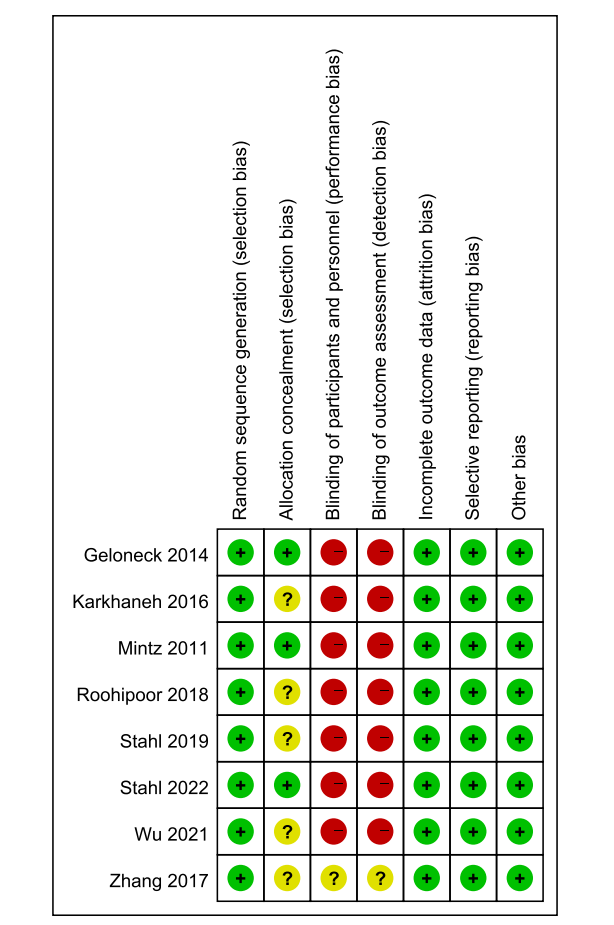


Fig. S1. Risk‐of‐bias summary of the included randomized controlled trial.

Table S2. Newcastle-Ottawa Risk of Bias Assessment of Non-Randomized Studies.

|  | Representativeness of the exposed cohort | Selection of the non-exposed cohort | Ascertainment of exposure to implants | Demonstration that outcome of interest was not present at start of study | Comparability of cohorts on the basis of the design or analysis | Assessment of outcome | follow up long enough for outcomes to occur | Adequacy of follow up of cohorts |
| --- | --- | --- | --- | --- | --- | --- | --- | --- |
| Sukgen | * | * | * | * | * | * | * | * |
| Arfat | * | * | * | - | - | * | * | * |
| Cheng | * | * | * | - | * | * | * | * |
| Blair | * | * | * | * | * | * | * | * |
| Erol | * | * | * | * | * | * | * | * |
| Chen | * | * | * | * | * | * | * | * |
| Jin | * | * | * | * | * | * | * | * |
| Milan | * | * | * | * | * | * | * | * |
| Elabbasy | - | * | * | * | - | * | * | - |
| Gunay | * | * | * | * | - | * | * | * |
| Hwang | * | * | * | - | * | * | * | - |
| Isaac | * | * | * | - | * | * | * | - |
| Kabataş | * | * | * | * | - | * | * | * |
| Kang | * | * | * | - | - | * | * | * |
| Kang | * | * | * | - | - | * | * | * |
| Kimyon | * | * | * | - | * | * | * | * |
| Lyu | * | * | * | - | - | * | * | * |
| Ekinci | * | * | * | - | - | * | * | * |
| Mueller | * | * | * | - | - | * | * | - |
| Murakami | * | * | * | - | * | * | * | * |
| Nicoară | - | - | * | - | * | * | * | * |
| Simmon | * | * | * | - | - | * | * | * |
| Riazi | * | * | * | * | - | * | * | * |
| Roohipoor | * | * | * | - | - | * | * | * |
| Sukgen | * | * | * | * | * | * | - | - |
| Sukgen | * | * | * | * | * | * | * | - |
| Su¨ren | * | * | * | - | * | * | * | * |
| Vujanović | * | * | * | - | - | * | - | * |
| Chen | * | * | * | * | * | * | * | * |
| Demir | * | * | * | - | - | * | * | * |
| Gunay | * | * | * | - | * | * | * | * |
| BJO¨ RN | * | * | * | - | * | * | * | - |
| Leng | * | * | * | * | - | * | * | - |
| Lin | - | - | * | * | * | * | * | * |
| Ling | * | * | * | - | * | * | * | * |
| Linghu | * | * | * | * | * | * | * | * |


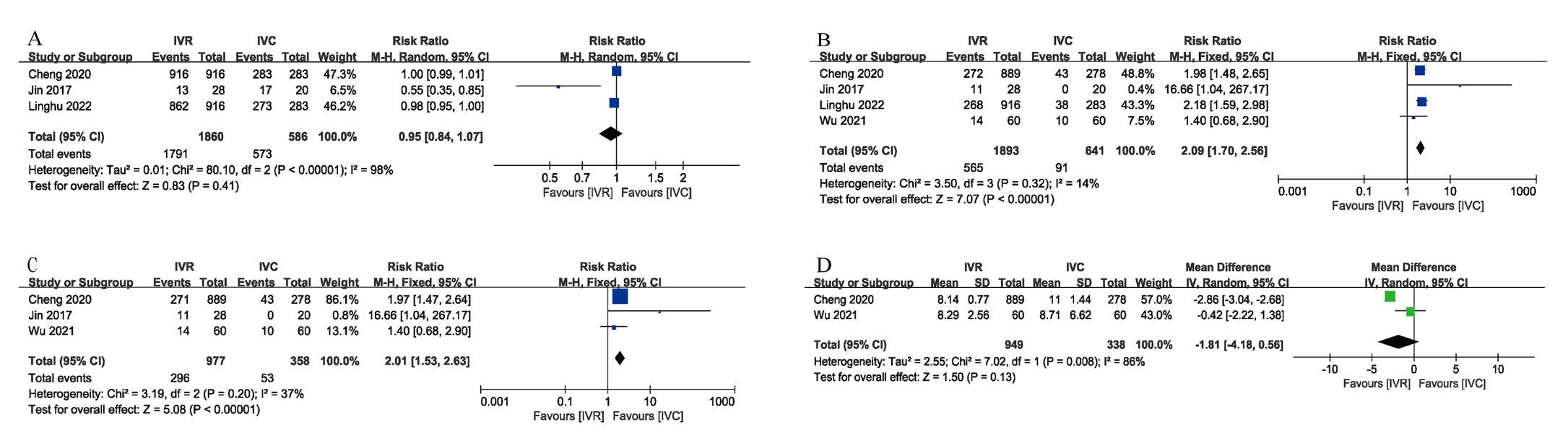


Fig. S2. Comparison outcomes between IVR and IVC.


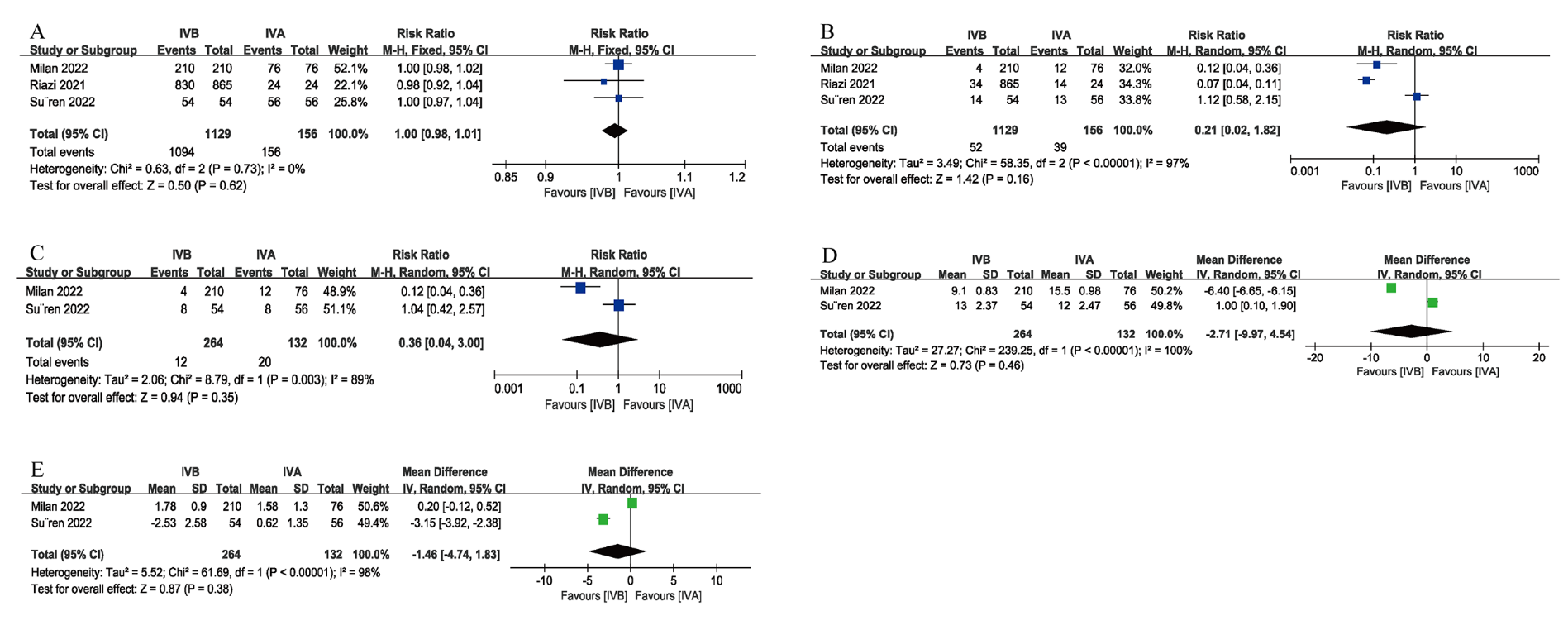


Fig. S3. Comparison outcomes between IVB and IVA.


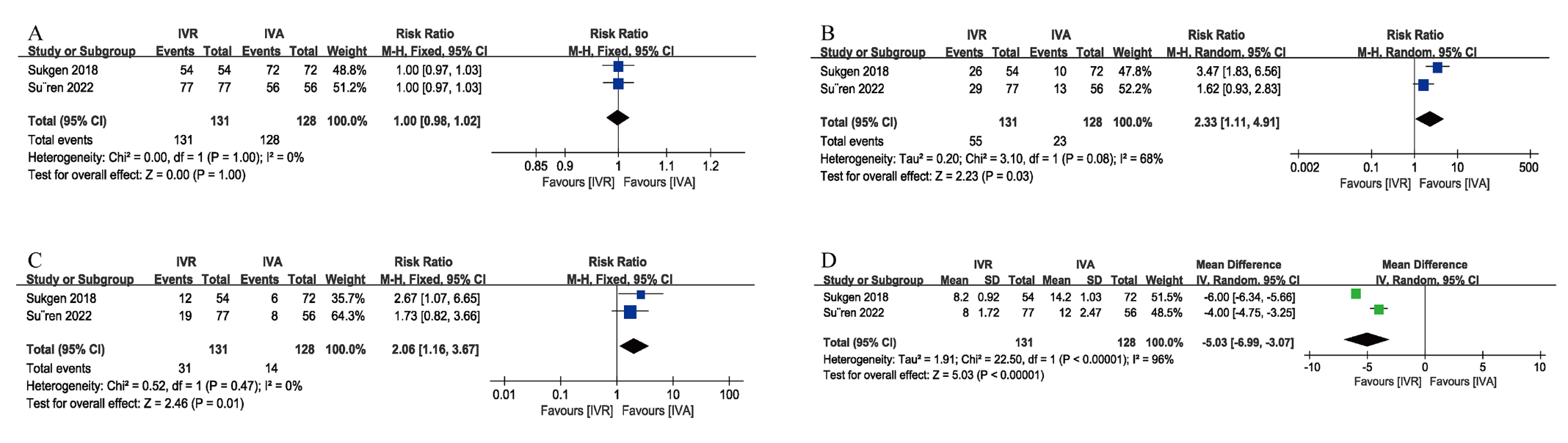


Fig. S4. Comparison outcomes between IVR and IVA.
